# Dental enamel bleached for a prolonged and excessive time: Morphological changes

**DOI:** 10.1371/journal.pone.0214948

**Published:** 2019-04-05

**Authors:** Kelly Fernanda Barbosa Vilhena, Bárbara Catarina Lima Nogueira, Nathalia Carolina Fernandes Fagundes, Sandro Cordeiro Loretto, Rômulo Simões Angelica, Rafael Rodrigues Lima, Mário Honorato Silva e Souza

**Affiliations:** 1 School of Dentistry, Institute of Health Sciences, Universidade Federal do Pará, Belém, Brazil; 2 School of Dentistry, Faculty of Medicine and Dentistry, University of Alberta, Edmonton, Canada; 3 Laboratory of Functional and Structural Biology, Institute of Biological Sciences, Universidade Federal do Pará, Belém, Brazil; 4 Institute of Geosciences, Federal University of Pará, Universidade Federal do Pará, Belém, Brazil; Queen's University at Kingston, CANADA

## Abstract

This work aimed to evaluate the roughness, microhardness, ultrastructure, chemical composition and crystalline structure in submitted teeth to a prolonged home bleaching regimen with 10% carbamide peroxide (10% PC) for different periods. The specimens were divided into the following groups: G1: negative control (application of water-soluble gel); G2: tooth whitening group (positive control), under application time recommended by the manufacturer (4h/14 days); G3: prolonged whitening 50%, under prolonged time recommended by the manufacturer in 50% (4h/21 days); G4: excessive whitening 100%, under exceeded manufacturer recommended time by 100% (4h/ 28 days). The results were evaluated descriptively and analytically. There were no changes in the roughness in any of the evaluated groups. However, the microhardness decreased in the G4 group. Scanning electron microscopy showed changes in the enamel surface of groups G2, G3 and G4. Dispersive X-ray spectroscopy identified changes in the concentration of chemical elements O, Mg, P, K in all groups. Thus, this study showed that prolonged home bleaching could cause changes in the ultrastructure, chemical composition and microhardness of the enamel.

## Introduction

The search for whiter teeth pattern has encouraged the consumption of bleaching agents. Due to this demand, a high diversity of products has been launched, with a portion of these characterized by low-cost sale without a dentist office visit [1]. Possible damages to the oral structure can occur with excessive use of bleaching products such as burns, transient sensitivity, reduction of microhardness and increase of roughness [2,3], information that is usually omitted by the manufacturer or even ignored by the population.

The bleaching gels act by breaking the unsaturated carbon bonds of the pigment molecules, making them smaller and less complicated, reducing the absorption capacity and increasing the light reflection and transmission [4].

Some factors are significantly associated with bleaching results, such as patient expectations, age, eating habits and oral hygiene. Although bleaching is effective, several authors have reported structural changes, including increased roughness, decreased microhardness and reduced enamel strength, decreased mineral content (calcium, phosphate and fluoride); as well as the appearing of clinical symptoms as gingival irritation and dentin hypersensitivity. The different types of peroxides with or without desensitizing or mineral repository are responsible for altering the chemical equilibrium of the enamel. These changes can result in structural alterations on the surface of the enamel when submitted to different concentrations of hydrogen peroxide. [4–7]. However, few studies have evaluated in a more detailed way the structure of dental enamel submitted to situations of bleaching for a long time.

The purpose of this study was to investigate the effects of prolonged dental bleaching on the different organizational levels of the dental enamel structure. Does the exposure of the enamel to the bleaching gel for a prolonged period promote any damage, loss and modification in the mineral and crystalline morphology of the enamel? For this analysis the study was designed using physical, chemical and ultrastructural evaluation methods that cover the organizational levels of dental enamel using the tests of roughness, microhardness, scanning electron microscopy (SEM), Energy-dispersive x-ray spectroscopy (EDS), X-ray diffraction (XRD).

## Material and methods

### Ethical aspects and sample definition

The Committee of Ethics in Research approved this study with Experimental Animals of the Federal University of Pará under the n° 83–2015. 116 bovine incisor teeth of the species *Bos taurus indicus* were used, being randomly divided for each analysis. Teeth erupted in the oral cavity and with a healthy crown were included in the study. Teeth with cracks or fractures were discarded.

After extraction, the teeth underwent a disinfection process, immersed in 0.1% thymol solution for one week. Then, the teeth were washed in running water, and any traces of blood or tissue were removed. Later, the teeth were analyzed in a stereoscopic magnifying glass (80x) to verify the healthiness of the buccal enamel on the coronal, middle portion. Finally, the teeth were stored in distilled water and kept at 4°C (within a maximum period of 30 days) until the tests were carried out, according to ISO/TR 11405:1994 guidelines [8].

### Teeth specimens

**Fig 1:**
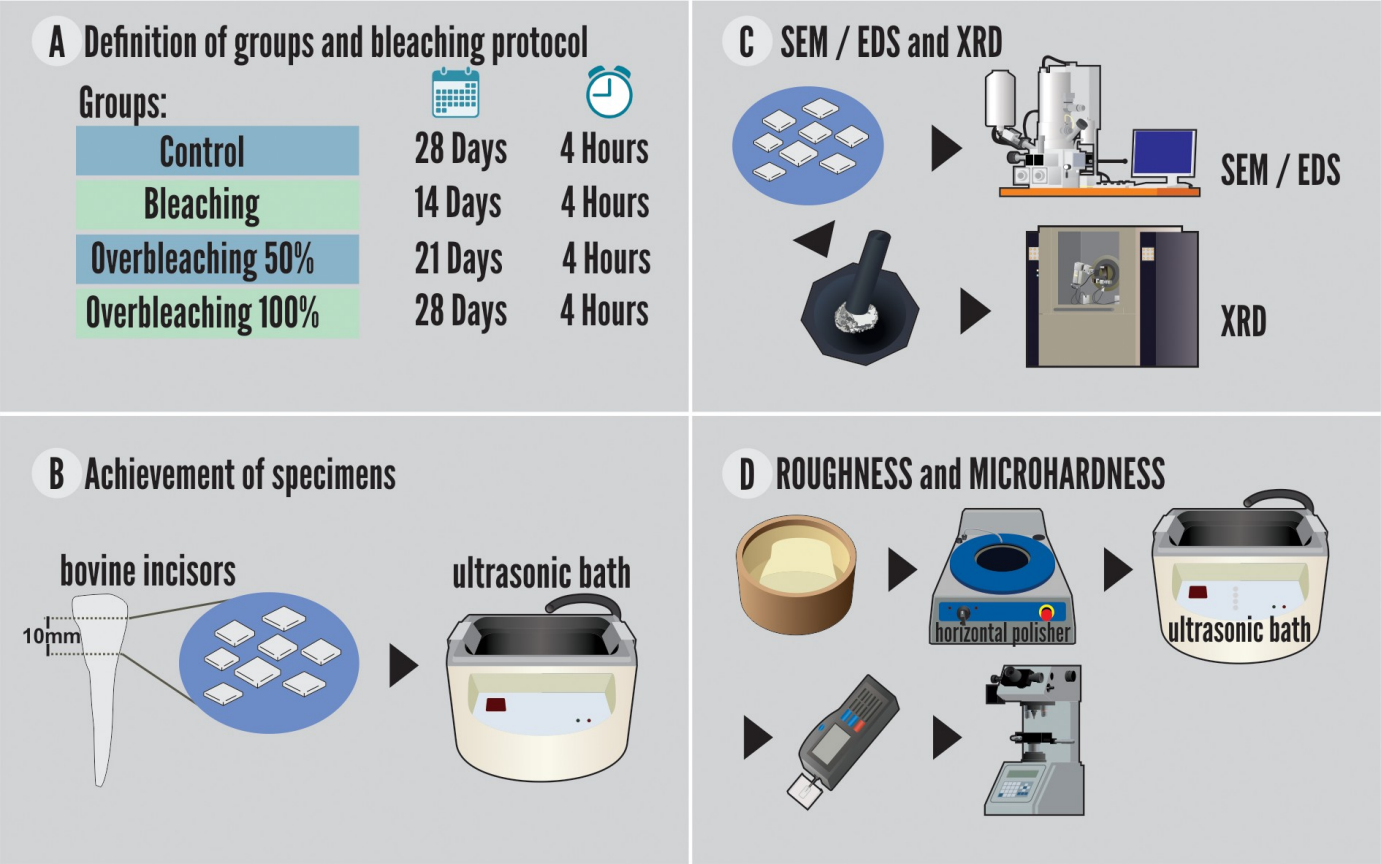
Preparation of the specimens and experimental steps.

To obtain the specimens, the teeth were sectioned transversely at two moments. The first cut was made with a laminated disk attached in a straight piece at a distance of 15 mm from the cementoenamel junction, measured with the aid of a digital caliper (DIN 862; Mitutoyo, São Paulo, Brazil), and parallel to the incisal edge. The second cut was performed at 5 mm from the cementoenamel junction, in the middle portion of the dental crown with a height of 8 mm. Then, longitudinal sections were performed in the mesiodistal direction to separate the buccal and lingual surfaces, and the samples were washed in an ultrasonic bath with distilled water for 2 minutes (Fig 1).

### Definition of groups and bleaching challenge

The bleaching agent used was 10% CP (Whiteness Simple 10%, FGM, Joinville, Brazil) as recommended by the manufacturer. Whiteness Simple Whitening Gel is composed by the following active ingredients: 10% Carbamide Peroxide and inactive ingredients (carbopol, potassium hydroxide, sodium fluoride, glycerol, deionized water).

The whitening protocol was defined based on a manuscript that used carbamide peroxide (CP 10%) to better control variables such as time, amount of bleaching agent and the use of artificial saliva. [9]. The enamel whitened for an extended period with CP 10% was increased by 50% and 100% in the application time recommended by the manufacturer.

The specimens were divided into groups, as follows:

- G1—Negative control: the surface of the enamel was treated with a water-based lubricant with a similar texture to the bleaching gel;
- G2 –Positive control: gel application according to the manufacturer's instructions (i.e., 4 hours a day for 14 days);
- G3 –Prolonged whitening 50%: application of the bleaching gel plus half the time of use recommended by the manufacturer. Application of the gel for 4 hours daily for 21 days.
- G4—Excessive whitening 100%: application of bleaching gel for twice the duration of use recommended by the manufacturer. Application of the gel for 4 hours daily for 28 days.

The application of the material was performed with a proportion of 0.1 ml of bleaching gel to 0.05 ml of artificial saliva, using acetate trays, made of a vacuum plasticizer and of a diameter compatible with the polyvinyl chloride matrix in which the teeth were fixed. This pattern was used to simulates the interaction between the carbamide peroxide and organic compounds present in the dental enamel when the bleaching gel is applied that occurs in a real clinical situation [9].

After application of the bleaching gel, the specimens were carefully washed with air/distilled water spray for 1min. Between the sessions, the specimens were stored in artificial saliva and conditioned again in a biological oven (37°C).

The composition of the artificial saliva was: sodium bicarbonate 2190mg, potassium phosphate 1270mg, magnesium chloride 125mg, calcium chloride 441mg, potassium chloride 820mg, sodium fluoride 4.5, nipazole 100mg, nipagin 10mg, sorbitol 24mg, carboxymethylcellulose 8mg, distilled water 1000ml (pH 7.0). The artificial saliva was manipulated in a local Pharmacy (A Fómula, Belém, Brazil).

### Roughness and microhardness

For the mechanical test (microhardness) and superficial evaluation (roughness), before the bleaching protocol, cylindrical molds of polyvinyl chloride with 20mm diameter and 1.5cm height were made. These molds were filled with acrylic resin and included the dental specimens with the free surface of analysis and face up. The buccal surfaces of the samples were sanded (#600, #800, #1200 and #2000) in a horizontal polisher and then washed in distilled water.

The microhardness and roughness tests were performed on the same specimens, adopting n = 20. The readings were performed in the following times: first roughness reading and microhardness measurement (T1): performed before the beginning of the whitening protocol (negative control); second roughness reading and microhardness measurement (T2): performed on the 14th day of bleaching (positive control); third roughness reading and microhardness measurement (T3): evaluated at the 21st day of bleaching; and fourth roughness reading and microhardness measurement (T4): performed at the 28th day of excessive bleaching.

The evaluation of the surface roughness (μm) was performed by a rugosimeter (SJ-301; Mitutoyo, California, United States of America). The parameter adopted was the arithmetic roughness (Ra) determined by the average (μm) of 3 readings, with a trace limit (Lt) of 5 mm and a sampling or cut-off length (La) of 0.25 mm.

The Knoop microhardness (KHN) was determined by a micro durometer (Future Tech FM 700; Future Tech Enterprise, Holdbrook, United States of America), with five indentations spaced 500μm with a load of 25g for the 20s. The indentations were performed parallel to the direction of the enamel prisms. The area of the enamel of the specimens was divided into four parts so that measurements of the microhardness were carried out in different parts.

### Ultrastructural analysis of the enamel surface

For scanning electron microscopy (SEM) analysis, specimens (n = 20) for each group were assembled in a sample holder and metalized for scanning electron microscopy (LEO-1430; Carl Zeiss, BW, Oberkochen, Germany). The metallization consisted of the vacuum precipitation of a micrometric film of a platinum alloy on the surface of the dental enamel. Electron micrographs of the enamel were obtained with a magnification of 1500x and were descriptively evaluated.

### Analysis of the chemical composition of the enamel surface

The chemical characterization of the enamel was done by EDS, Scanning Electron Microscope Tool (LEO-1430; Carl Zeiss, BW, Oberkochen, Germany). The specimens were assembled into stubs and metalized with a micrometric platinum alloy film. The concentrations by weight of the following chemical elements were evaluated: Ca (Calcium), O (Oxygen), P (Phosphorus), Mg (Magnesium), K (Potassium), Ti (Titanium), V (Vanadium), Zn (Zinc), Cl (Chlorine) and Zr (Zirconium). Among these elements are the main forming elements of the crystals of hydroxyapatite [Ca_10_(PO_4_)_6_(OH)_2_]. The same specimens were used for SEM and EDS.

### Crystallography analysis of the enamel

The X-ray diffraction analysis was used to determine the crystal of the dental enamel. For this analysis, four enamel fragments were used, with an average weight of 1g per group. After completion of the bleaching protocol and the control, the dental fragments were removed from the silicone matrices and dried in a biological oven (37°C) for 24 hours. The dental fragments were crushed using a pestle and mortar, which resulted in a powder form sample. The obtained powder of each group (G1-G4), were taken to perform the analyzes of X-ray Diffraction. The X'Pert Pro 3 MPD (PW 3040/60) PANalytical x-ray diffractometer with PW3050 / 60 goniometer (θ-θ) and Cu Anode X-ray tube (Kα1 = 1.540598 Å) model LFFDK401283, long thin focus, Kβ Ni filter, Pix Cel1D (Real Time Multiple Scanning) detector in the scanning mode and with active length 2,122°. The following instrumental conditions were used: scanning 4° to 70° 2θ, 40 kV, 40 mA, step 0.02° in 2θ and time/step of 20s, fixed slot 1/2 and anti-spreading 1°, mask 10 mm, spinning sample movement with 1 rps. Data was read using X'Pert Data Collector 2.1a software (PANalytical, São Paulo, SP, Brazil) and data processing with X'Pert HighScore 3.0 software (PANalytical, São Paulo, SP, Brazil).

#### Data analysis

The electron micrographs obtained by SEM were evaluated descriptively, observing the variations in the micromorphology of the dental enamel of the different groups analyzed. The results obtained by the EDS, microhardness and roughness tests were submitted to the normality test (Shapiro-Wilk test) and evaluated using the one-way Variance Analysis (ANOVA) with Tukey post-test. The p≤0.05 level of significance was adopted for all the analyzes undertaken. All statistical analyzes were performed on GraphPad Prism software (GraphPad, San Diego, United States of America).

## Results

### Enamel roughness

Exposure of the enamel to the gel for different periods of time did not result in changes in the surface roughness of the bleached enamel when compared to the control group (p = 0.0752) (Table 1).

**Table 1:** ANOVA applied to the mean (and standard deviation) of the surface roughness (μm) for the different times of application of 10% carbamide peroxide.

	Times of Application			
	T1	T2	T3	T4
Mean (Standard deviation)	1.0345 (±0.0073)^a^	1.0376 (±0.0051)^a^	1.039 (±0.0091)^a^	1.0397 (±0.0094)^a^

Different letters indicate statistical difference at 5%.

T1, control group; T2, 14 days of CP 10% application; T3, 21 days of CP 10% application; T4, 28 days of CP 10% application.

### The interference of the increase of the time of use of the bleaching gel on the enamel microhardness

The results showed significant values of reduction of G4 enamel microhardness (194.96 ± 37.45) when compared to the other groups in all evaluated period (Table 2).

**Table 2:** ANOVA applied to the mean (and standard deviation) of the surface roughness (μm) for the different times of application of 10% carbamide peroxide.

	Times of Application			
	T1	T2	T3	T4
Mean (Standard deviation)	244.14 (±44.12)^a^	234.86 (±49.21)^a^	219.86 (±30.51)^a^	194.96 (±37.45)^b^

Different letters indicate statistical difference at 5%.

T1, control group; T2, 14 days of CP 10% application; T3, 21 days of CP 10% application; T4, 28 days of CP 10% application.

### Ultrastructural alteration of the surface of enamel whitened for a prolonged time

**Fig 2:**
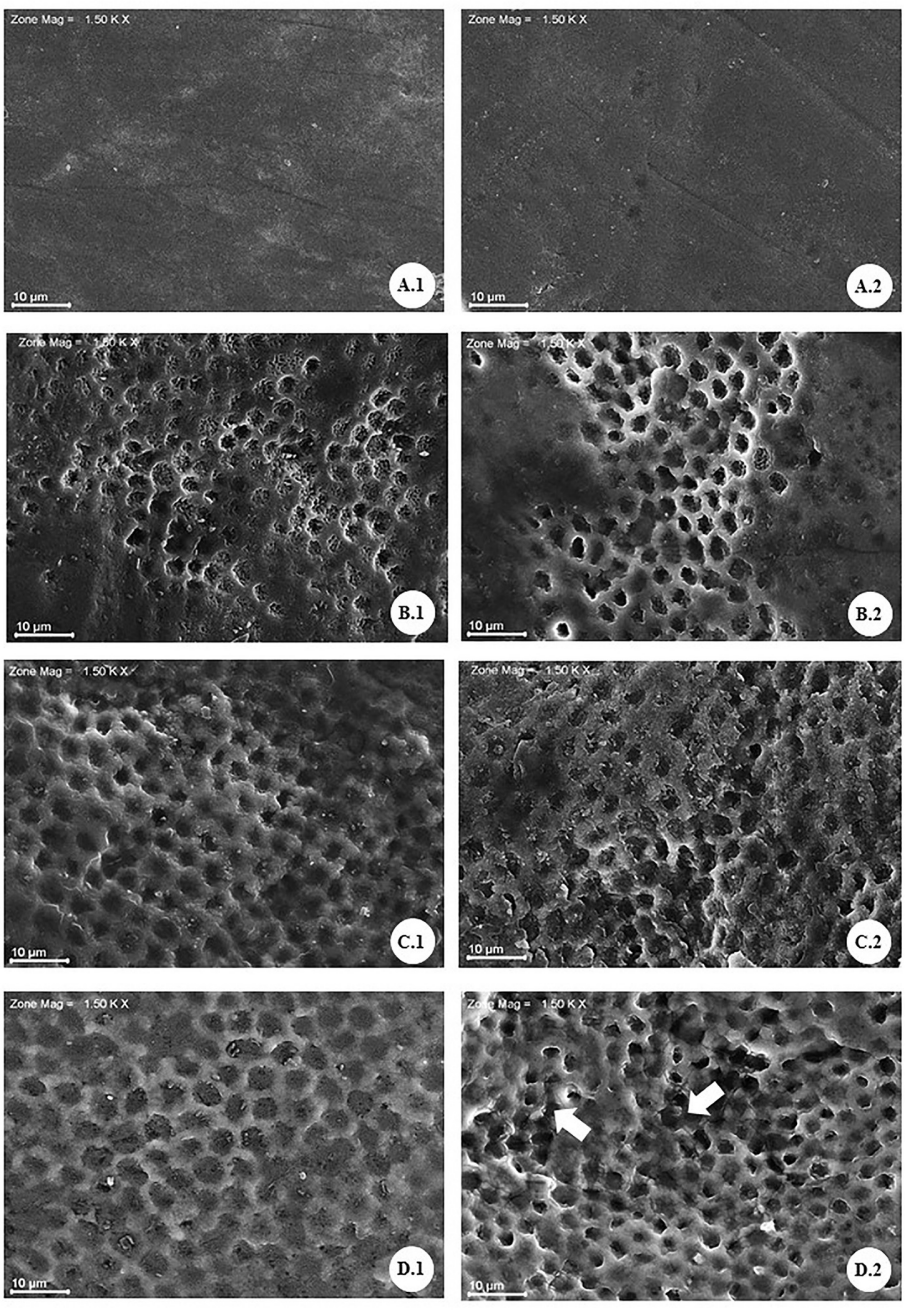
Electron micrographs of the enamel surface submitted to different bleaching times with 10% carbamide peroxide (PC 10%). A1 and A2: unclarified enamel (negative control; G1), the absence of changes in enamel surface, the presence of aprismatic layer. B1 and B2: bleaching with PC 10% for 14 days (G2), indicated partial removal of the prismatic layer and gradual exposure of the prismatic enamel. C1 and C2: PC 10% for 21 days (G3), showed a continuity of removal of the aprismatic layer and greater exposure of areas of prismatic enamel. D1 and D2: PC 10% for 28 days (G4), show a presence of an irregular surface with more significant loss of the central portion of the prisms (D.1) and discontinuity of the interprismatic enamel (arrows) (D.2).

The electromicrographs show images of the enamel surface of groups (G1, G2, G3 and G4) in the different times of bleaching proposed by this study. In group G1 (Fig 2A1 and 2A2), the presence of aprismatic enamel was observed, preserving its histological structure. After 14 days of bleaching (G2) (Fig 2B1 and 2B2), the beginning of changes in the surface of the enamel bleached, with a slight exposure of the prismatic enamel, loss of the central portion of the prisms and maintenance of the limits interprismatic morphological changes become more evident in the G3 group (Fig 2C1 and 2C2), with larger areas of exposure of the interprismatic enamel. For group G4 (Fig 2D1 and 2D2), the same changes were observed, however, in a more severe manner and increased the loss of continuity of the interprismatic enamel.

### Analysis and modifications observed in the levels of chemical constituents present on the enamel surface

The chemical composition of dental enamel was analyzed in all groups. The results showed, statistically, significant alterations in the levels of chemical constituents O, Mg, P, and K presented in Table 3.

**Table 3:** ANOVA and post-test of Tukey, adopting level α of significance (p≤0.05), applied to the variations of the concentration values by weight (p%) of the chemical elements observed by means of spectroscopy of dispersion of rays- x (EDS) of dental enamel submitted to different bleaching times with 10% carbamide peroxide.

	G1	G2	G3	G4
O	18.7924 (±5.5566)^a^	21.2522 (±5.5534)^b^	23.1655(±6.0886)^b^	23.6205 (±6.2071)^b^
Mg	0.4251 (±0.2184)^a^	0.2070(±0.1398)^b,c^	0.2708(±01763)^b^	0.1659(±0.1068)^c^
P	15.996 (±1.0107)^a^	15.4138 (±1.0813)^b^	15.2069 (±1.0787)^b^	14.4781(±0.9053)^c^
Cl	0.0496(±0.0446)^a^	0.0444 (±0.0443)^a^	0.0487 (±0.0692)^a^	0.0459 (±0.0718)^a^
K	0.0793(±0.0310)^a^	0.0736 (±0.1803)^a^	0.0613(±0.0434)^a^	0.0578 (±0.1110)^c^
Ca	42.0321(±6.3343)^a^	43.5024 (±4.2011)^a^	44.7616 (±4.0534)^a^	41.5911 (±4.6041)^a^
Ti	0.0964(±0.0316)^a^	0.1053(±0.0587)^a^	0.1131 (±0.0589)^a^	0.0888 (±0.0401)^a^
V	0.1514(±0.0243)^a^	0.0975 (±0.0323)^a^	0.1052 (±0.0533)^a^	0.0944 (±0.0418)^a^
Fe	0.4088(±0.1001)^a^	0.1490(±0.0511)^a^	0.1439 (±0.0486)^a^	0.1312 (±0.0415)^a^
Zn	0.5687(±0.1077)^a^	0.4089(±0.1292)^a^	0.4513 (±0.2002)^a^	0.3724 (±0.0951)^a^
Zr	23.1655(±2.9383)^a^	18.2957 (±1.7325)^a^	18.9732 (±3.0090)^a^	18.8083 (±1.7587)^a^

Different letters indicate statistical difference at 5%.

G1, 28 applications of water-soluble gel; G2, 14 days of CP 10% application; G3, 21 days of CP 10% application; G4, 28 days of CP 10% application.

### Crystallographic conservation of the enamel

**Fig 3:**
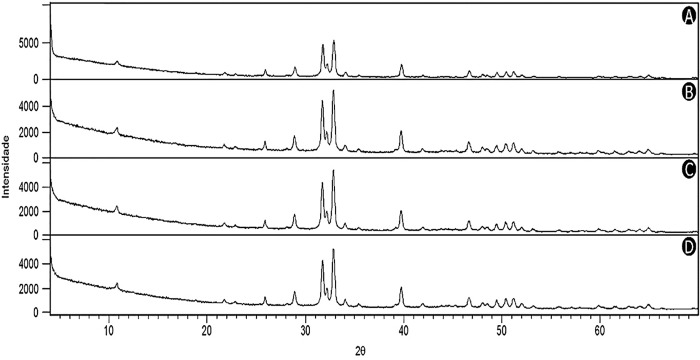
Diffractograms of the respective experimental groups G2 (B), G3 (C) and G4 (D) showed similarity to the control group G1 (A), which characterizes the hydroxyapatite crystal (Hap). The coincidence of the peaks in the graph according to the different time periods of the bleaching indicate to be the same crystal.

The interpretation of the diffractograms (Fig 3) of the experimental and control groups showed that the hydroxyapatite crystals maintained an organizational pattern of the mineral structure in all the analyzed groups.

## Discussion

The use of 10% CP gel for an extended period was able to cause changes in the structure and microhardness of the dental enamel, but without changes in the enamel roughness (Table 1). The O, Mg, P, K elements varied in their levels throughout the studied period without modification of the crystalline structure of the enamel.

The bovine model used in this study is a valid method in the scientific environment as a substitute for the use of human teeth in several studies. The physical, chemical and ultrastructural properties of bovine and human enamel were evaluated in several comparative studies of the most varied analyzes, and similarity of the structure have been reported [10–12].

The surface roughness of dental enamel did not change in the evaluated groups. The results of the roughness test are in agreement with some other studies, which did not observe significant alterations in the roughness between the bleached and unclarified surface using 10% carbamide peroxide according to the manufacturer's recommended protocol [13,14].

Carbamide peroxide may promote changes in the mechanical properties of the enamel due to the action of its by-products, such as urea and oxygen. Free radicals of hydrogen peroxide, a component in which carbamide peroxide degrades, have no specificity and react with and degenerate the dental tissues [14,15]. Other studies that used CP 10%, following the manufacturer's norms or the standard application time, verified changes in the microhardness values of the bleached enamel. The decrease in the microhardness values is attributed to peroxide action on the enamel organic matrix, since the organic matrix is degraded, resulting in a more fragile enamel [16–18]. Thus, the abusive use of the bleaching gel for a prolonged period promotes a higher degradation of the organic matrix of the enamel, compromising the structure, leaving it more fragile, an aspect identified by the decrease of the microhardness values in the group G4.

As observed, there was reduction of microhardness and absence of changes in surface roughness. The initial hypothesis was that with the reduction of the microhardness there was an increase in the roughness. However, the bleaching agent alone was not able to promote roughness change. We can infer that the association of whitening with elements such as brushing, food friction and diet can be related to enamel surface roughness changes. [19,20]

The SEM results revealed changes in the ultrastructure of bovine dental enamel after prolonged use of CP 10% gel. The images obtained with a magnification of 1500x were analyzed descriptively. The electromicrographs of the control group (G1) evidences the normal histological pattern characteristic of the surface of bovine dental enamel. It was observed an intact surface marked by the presence of the aprismatic enamel, characteristic of the pattern of this external morphology, without any modification [18,21,22].

In G2, besides the partial loss of the aprismatic layer, a pattern compatible with Silverstone's type I demineralization was observed, probably due to the action of the peroxide on the surface of the tooth identified by the loss of the central portion of the prisms [23]. In G3, the electron micrographs evidenced the progression of the demineralization process, due to the greater involvement of the central portion of the enamel prisms, as well as the increased exposures of prismatic enamel areas. In the evaluation of the G4 images, the increase in the severity of the changes in the surface treated by the period of 28 days was noticed.

The pattern observed in the SEM images for the groups bleached for an extended period was compatible with the enamel demineralization patterns when conditioned by phosphoric acid, more specifically the Silverstone type III pattern [24]. In this pattern, the involvement of the peripheral portion of the crystals within the prisms, discontinuity of interprismatic enamel and the irregular surface are predominantly observed. This characteristic has been evidenced in the bleaching treatments when concentrations compatible with the office treatment, such as the Hydroxide Peroxide 35% [25] were used, and no such alteration was observed when home treatment was performed at the time recommended by the manufacturer. Corroborating these data, we can mention the characteristics by this study described in groups G1, G2 and G3.

The EDS, an analytical technique used to characterize chemical elements, revealed statistically significant differences in O, Mg, P, and K levels in the different experimental groups. Regarding Oxygen, there was an increase in its concentration over the application period of the bleaching gel. The increase in oxygen levels is probably related to the action of peroxide on the mineralized enamel structure, a reaction that is well mentioned in the literature [2,18,23,26]. The increase of the oxygen levels can compromise the adhesive properties of the interface between the dental substrate and the restoration, due to the higher difficulty of polymerization caused by the contact of the oxygen with Carbon present in the monomer molecule, thus compromising the quality of enamel hybridization cleared [27,28]. Besides, a decrease in Mg levels was observed as a function of the bleaching agent application time. The reduction of this element can be related to the fact that it is among the first elements to be removed during the reaction process of the peroxide with the dental surface. Thus, this phenomenon may be indicative of a demineralization process in bleached enamel [29].

Numerically, there was a decrease in Ca levels when comparing the G4 and G1 groups, although no statistically significant difference was identified. Regarding P, a statistically significant decrease was detected concerning the other groups (G1, G2 and G3), highlighting a reduction in the levels of these two elements. In another study [16], using CP 10%, a decrease in Ca concentration levels, as well as in Ca and P ratio, was observed for bleached enamel. The relationship between Ca and P is an essential indicator of the remineralization process [29,30]. The decrease in the level of the two elements could lead to an irreversible alteration, preventing the remineralization process from occurring. Naturally, other characteristics of the medium, such as pH, would also have influence, aspects not included in the present study must be evaluated for a more accurate answer to this topic.

Similarly, the levels of concentration of the K ion decreased during the study time. The role of K has not yet been fully elucidated, the reduction of K levels was compatible with those observed in the study by Cakir and Castro [29,30]. It was suggested that the decrease in K could be related to a reduction in the organic compounds of the enamel.

The presence of these elements may be related to endogenous factors, during the formation of the enamel organ, or even exogenously, through the incorporation posterior to the formation of the enamel, which is absorbed, being solubilized in the buccal environment and impregnating the dental structure due to eating habits [26,31].

Analysis of the diffractograms showed that the G1, G2 and G3 and G4 groups had the same mineral pattern—Hydroxyapatite (Ca_10_(PO_4_)_6_(OH)_2_). This result was compatible with the findings of Oltu and Wang [32] that observed the crystalline structure under whitening condition combining concentration, bleaching agents and time and obtained absence of an alteration in the crystalline configuration of the enamel.

When we confront the variations observed for element levels, especially Ca and P, and the findings found using x-ray diffraction analyzes, we noticed an incongruence, that is, there was a change in the levels of Ca and P elements, but there was no modification of the crystal structure of the enamel. Regarding this result, a question was established: “how can we identify the isolated variation of chemical elements and not of their crystalline molecular structure ?”, After all the chemical elements are not loose but united in molecules. One explanation would be that the observed changes may be indicating and reinforcing the idea of reversible changes, there is a change in the mineral surface, but it is transient and remineralizing, so it does not compromise the molecular structure of the enamel prism.

It is also worth mentioning that in the present study, the specimens were left immersed in artificial saliva throughout the experiment, which probably contributed to reverse the demineralization process, that is, remineralization occurred and there was no partial loss of structure.

The phenomenon of remineralization is entirely feasible in the oral environment due to the well-known biochemical aspects [25– 33–34], but the need for specific biochemical situations to be present, which is more wholly found in the oral cavity [29– 34]. Another factor to be considered relates to the composition of bleaching gels. The 10% CP used in this study has a desensitizer in the composition, the sodium fluoride. These agents could also aid in the reversal of demineralization processes [6,7,18], as found mainly in group G4.

Analyzing the results together, we can understand that there was a progression of damage to the mineralized structure of the enamel, perhaps irreversibly. On the other hand, it would be necessary to carry out more specific tests to prove this fact. Repeating the tests proposed in the present study waiting for a few weeks' post bleaching and leaving the specimens immersed in artificial saliva for a few weeks could also clarify some aspects not well determined. Moreover, there is a contact of bleaching gels with soft tissues, such as mucous membranes and even the pulp connective tissue, which could exacerbate eventual damage to these tissues [1,3,4,26].

Thus, based on the results of this study, we can say that the excessive use of CP 10% for periods of up to 28 days causes some changes in the Macro and the Enamel Ultrastructure, for too long there is a change in the surface of the enamel that can mean damage irreversible to the mineral structure by the incorrect use of the exceeded time used of the bleaching agent even at low concentration.

Besides, it can be understood as the contribution of this research the presentation and discussion of data obtained through more specific tests related to the intimacy of the enamel structure, data little mentioned in the pertinent literature.

## Conclusion

Finally, the results of this investigation show that the 10% PC bleaching gel, when used in a way that exceeds the time recommended by the manufacturer, was able to reduce the microhardness, modify the ultrastructure and promote variations in the chemical composition of the enamel.
